# Characterizing the antioxidant and antifungal properties of nano‐encapsulated pistachio hull extract in fenugreek seed gum to maintain the quality and safety of fresh pistachio

**DOI:** 10.1002/fsn3.4209

**Published:** 2024-05-13

**Authors:** Ali Fatemi, Ali Najafi, Razie Razavi, Shima Jafarzadeh

**Affiliations:** ^1^ Department of Food Science and Technology, Damghan Branch Islamic Azad Unversity Damghan Semnan Iran; ^2^ Department of Food Science and Technology Sari Agricultural Sciences and Natural Resources University Sari Mazandaran Iran; ^3^ Centre for Sustainable Bioproducts Deakin University Waurn Ponds Victoria Australia

**Keywords:** nanoemulsion, phenolic compounds, pistachio oil oxidation, release, shelf life

## Abstract

The quality of pistachio, one of the export products of Iran, will be decreased during storage as a result of mold spoilage, toxins production, and oil oxidation. This study aimed to investigate the capability of pistachio hull extract (PHE) loaded in fenugreek seed gum (FSG):whey protein isolate (WPI) nanoemulsion to control oil oxidation, and fungi growth in fresh pistachio nut during storage at 4°C. The total anthocyanin and total phenolic content of the PHE were 125.44 μg/g and 675.18 mg/g, respectively. The DPPH radical scavenging activity of PHE at 100 ppm was higher than that of tert‐butylhydroquinon (TBHQ). In comparison with other concentrations, 50 ppm showed the strongest antifungal activity against *Aspergillus flavus*, *Aspergillus parasiticus*, and *Aspergillus nomius*. All nanoemulsions have a mean size lower than 265 nm. The polydispersity index (PDI) of different nanoemulsions was lower than 0.3, and a negative zeta potential was observed. The encapsulation efficiency was higher than 67.0% and all nanoemulsions had spherical morphology. The pistachio nuts were coated with different coating solutions containing 0 and 100 ppm of PHE and stored at 4°C for 8 weeks. The results showed that the pistachio sample coated with a composite coating of WPI and FSG containing 100 ppm of PHE has a higher moisture content and lower changes in L*, a*, and b* indexes, oil oxidation, fungi development, and total mold and yeast count. This treatment exhibited higher overall acceptance than other samples at the end of storage time. The results of this study suggest the use of biodegradable coatings enriched with natural extracts that have high antioxidant and antifungal activities.

## Graphical abstract

This research is about natural antioxidant and antimicrobial agents in pistachio hull extract incorporated in edible coatings to increase the shelf life of fresh pistachio.

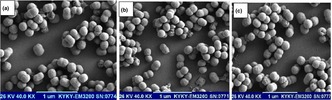

## 1 INTRODUCTION

Pistachio (*Pistacia vera* L.) is one of the most important trade‐worthy nuts widely cultivated in dry, saline, and hot areas like Iran. This nut is a good source of minerals, tocopherols, and bioactive compounds like anthocyanins, flavonoids, and lutein (Gheysarbigi et al., 2020; Tavakolipour et al., 2020). In the food industry, pistachio nuts are used to produce pistachio butter and cakes. Dried pistachio nut contains 2.5% ash, 4% moisture, 15% fiber, 22% protein, and 55% fat (Tavakolipour et al., 2020).

*Aspergillus flavus*, *Aspergillus parasiticus*, and *Aspergillus nomios* are the most important fungi found on different nuts, especially pistachio, which can produce aflatoxin B_1_, B_2_, G_1_, and G_2_. Produced aflatoxins lead to cancer in internal organs such as the liver and digestive system. Factors such as high moisture content or storage in high relative humidity lead to the production of a high content of aflatoxin in pistachio nuts. The European Committee of Standards has approved the limitation of aflatoxin in pistachio nuts at 10 ng/g (Bensassi et al., 2010).

Using synthetic packaging and low temperatures is the most recommended solution to prevent the growth of fungi and lipid oxidation in nuts. The food industry is the principal consumer of synthetic plastics that cause environmental pollution. The use of cooling equipment during the storage period and supply chain is difficult and expensive. Therefore, the storage of nuts at room temperature can be very beneficial (Khoshnoudi‐Nia & Sedaghat, 2019).

Edible coatings are thin layers composed of proteins, carbohydrates, lipids, and bioactive compounds that can be applied directly to the food. These coatings regulate moisture content and possess the potential to function as carriers for numerous bioactive compounds, such as natural antioxidants and antimicrobials. Edible coatings boost the effectiveness of natural food preservatives and limit microbial growth on the food surface (Yaashikaa et al., 2023).

The use of active packaging and coatings as a new part of food packaging provides more protection than conventional packaging to conserve the stability, and quality of food. In practice, edible coatings and active packaging play a protective role by creating a semi‐permeable barrier against the transfer of molecular components (such as moisture, gases, and flavors) and controlling adverse reactions responsible for undesirable changes in food products. Edible coatings can usually be considered as a secondary packaging system to reduce the need for synthetic packaging materials. In other words, edible polymers can be consumed together with food products, reducing environmental concerns about the depletion of natural resources and the waste problems of synthetic packaging materials, especially plastics. Therefore, edible polymers are considered environmentally friendly and sustainable packaging materials (Estrada et al., 2019).

An edible coating can act as a carrier for bioactive components. In addition to their antioxidant and antimicrobial properties, these compounds can increase the nutritional value of food products (Murmu & Mishra, 2017). Therefore, the use of edible coatings containing natural extracts is the main priority of consumers due to the absence of harmful compounds and also due to their abundance, environmental friendliness, and availability. Edible coatings create a layer between the product and the atmosphere. They lead to a reduction in moisture loss, preventing oxygen penetration and the growth of microorganisms and reducing the transfer of moisture between the product and the surrounding environment (Hashemi et al., 2021).

Complex biopolymers based on proteins and carbohydrates have excellent barrier properties against oxygen penetration due to their special molecular structure. Incorporating natural preservative compounds in edible coatings leads to the controlled release of antimicrobial and antioxidant compounds, which ultimately improves the quality and stability of the coated food (Hashemi et al., 2021).

Gums are used to describe a group of natural polysaccharides that have wide industrial applications. In recent years, the demand for seed gums in food, cosmetic, and pharmaceutical applications has increased significantly (Dhull et al., 2020). Gums have high viscosity and mucilage, form a complex with proteins, and are highly efficient at trapping extract compounds. They also contain antioxidants and antimicrobial compounds. Fenugreek (*Trigonella foenum*‐*graecum*) is an annual plant that belongs to the leguminous crop. The main polysaccharide in fenugreek seed endosperm is galactomannan, which has various applications in food, medicine, cosmetics, and health products in the production of tablets, encapsulation coating material, and packaging coating. They have increased the consistency and stability of emulsion and suspension systems. They can also be used as dietary fiber and fat substitutes (Dhull et al., 2020). Whey protein is a byproduct of cheese factories and is used as an additive in many processed foods, such as confectionery, bakery, ice cream, and baby food. By producing edible coatings from whey protein, the nutritional value and shelf life of coated food will be improved (Delfanian et al., 2018).

Encapsulation of plant extracts using biopolymer coatings can increase the stability and shelf life of encapsulated materials by protecting them from environmental, enzymatic, and chemical changes, providing a buffer state against pH changes, dealing with thermal and ionic changes, protecting against unpleasant tastes and odors, and controlled release of encapsulated material (Manojlović et al., 2010). There are various methods for encapsulating extracts, the most important of which is the use of emulsion production methods (McClements et al., 2009).

In recent years, the use of natural antioxidants extracted from agricultural and industrial by‐products has increased due to their stability, high antioxidant activity, and non‐toxicity. These extracts can be used as substitutes for synthetic antioxidants, color, and oxidative stabilization (Tabaraki & Ghadiri, 2016). Green pistachio hulls make up about 40% of the pistachio weight. These by‐products contain significant amounts of phenolic compounds. Considering the high volume of pistachio hull by‐products and the presence of phenolic and antioxidant compounds, it is possible to improve their shelf‐life quality by extracting the mentioned compounds and using the extract to coat fresh food products (Rafiee et al., 2017; Seifzadeh et al., 2018).

The unsaturated fatty acids in pistachio lipids have made them sensitive to oxidation and toxic fungi growth. Several studies have been conducted to investigate the phenolic compounds and antioxidant activity of pistachio hull extract (PHE) (Seifzadeh et al., 2019; Tabaraki & Ghadiri, 2016) and the use of different edible coatings like whey protein concentrate (Khoshnoudi‐Nia & Sedaghat, 2019), gelatin (Saeedi et al., 2022), Arabic gum (Hashemi et al., 2021), methyl cellulose (Moslehi et al., 2021) and carboxymethyl cellulose on pistachio (Tavakolipour et al., 2020) and hazelnut (Razavi et al., 2021). Since no research has been conducted on the antioxidant and antimicrobial effects of encapsulated PHE in whey protein isolate and fenugreek seed gum to increase the shelf life of nuts, for the first time, this research will be conducted to investigate the antifungal and antioxidant properties of PHE loaded in fenugreek seed gum and whey protein isolate to increase the shelf life of pistachio nuts. This research is completely novel because, in this study, the natural bioactive compounds with antioxidant and antimicrobial properties will be extracted from pistachio hull waste, which is produced in large quantities every year. Also, fenugreek gum has been extracted for the first time and used for the encapsulation of the pistachio hull extract. Using the results of this research, the shelf life of coated fresh pistachios can be increased.

## 2 MATERIALS AND METHODS

### 2.1 Materials

Ahmad Aghaei variety pistachio was collected from a pistachio garden in Damghan in September 2022. Fenugreek seeds were purchased from the local market. The chemicals used in the research were analytical grade and obtained from Sigma Aldrich Company, and the desired fungi were obtained from the microbial collection of the Scientific and Industrial Research Organization of Iran. Whey protein isolate was also obtained from Merck, Germany.

### 2.2 Methods

#### 2.2.1 Extraction of PHE

The pistachio hulls were separated by hand and dried in an oven at 40°C for 4 h. Then they were turned into a powder with an approximate particle size of 0.5–2 mm. To obtain PHE, distilled water was added to dried powder at a ratio of 15:1. Extraction was done using a shaker for 8 h at room temperature. Then it was centrifuged at 3000 *g* for 15 min. The supernatant was separated using filter paper (Seifzadeh et al., 2019).

#### 2.2.2 Measurement of the bioactive compound content and antioxidant activity of the PHE extract

The total anthocyanin and total phenolic content of the PHE extract were measured according to the method described by Saeedi et al. (2022). For this purpose, 1 g of pistachio hull powder was mixed with 10 mL of hydrochloric acid solution 0.01% w/v in methanol using an ultrathorax homogenizer for 1 min and then centrifuged for 10 min at 4°C and 10,000 *g*. The absorbance was read using a UV–vis spectrometer at 510 and 700 nm at pH 1 and 4.5. To measure the total phenolic content, 1 gr of pistachio hull was mixed with 10 mL of methanol 80% (v/v) for 1 min using an ultrathorax homogenizer, and then it was centrifuged for 10 min at 4°C and 10,000 *g*. Then, 250 μL of the extract was mixed with 50 μL of Fulin Ciocaltio reagent, and 2 mL of distilled water was added to it and stirred for 3 min. After 3 min of storage at room temperature, 250 μL of a saturated sodium carbonate solution was added to it and kept at 37°C for 30 min. The absorbance value was read at 760 nm. The total anthocyanin and total phenolic content were obtained based on the relevant equations. The antioxidant activity of the extract was measured using the DPPH radical scavenging method (Saeedi et al., 2022).

#### 2.2.3 Measurement of the antifungal activity of PHE extract

The method of Saetae and Suntornsuk (2010) was used with a slight modification. *Aspergillus flavus*, *Aspergillus parasiticus*, and *Aspergillus nomius*, which are the most common pistachio fungi, were chosen as indicators. Fungi were cultured on potato dextrose agar medium and incubated for 7 days at 25°C. A commercial synthetic antifungal, TBHQ (100 ppm), was also considered a positive control. Different concentrations of PHE were added to the potato dextrose agar medium and incubated for 7 days at 25°C. Various studies have proven the antifungal effects of TBHQ, especially in inhibiting aflatoxin. Therefore, for positive control, a commercial synthetic TBHQ was used. The diameter grown by fungi in a petri dish was measured and reported as a percentage of antifungal activity (Saetae & Suntornsuk, 2010).

#### 2.2.4 Extraction of fenugreek seed gum

Fenugreek seeds were cleaned by hand to remove foreign materials, and seed gum was extracted by the method of Kutlu et al. (2020) under optimal conditions (water to seed ratio 1 L:100 g, temperature 60°C, and pH = 7). The gum was separated from the swollen seeds by passing the seeds through an extractor equipped with a rotating plate. After passing through a vacuum filter, the obtained solution was filtered to remove excess particles and then dried in an oven at 45°C (Kutlu et al., 2020).

#### 2.2.5 Preparation of nanoemulsion‐loaded PHE

Whey protein isolate (WPI) solution (4% w/v) was prepared in phosphate buffer with a pH = 6 and kept in the refrigerator for 24 h. A fenugreek seed gum (FSG) solution (2% w/v) was prepared in distilled water. To prepare a nanoemulsion, 7% of PHE at 100 ppm concentration was added to the emulsion phase, which contains 25% Span 80 and 68% soybean oil. The mixture was stirred with a magnetic stirrer at 1000 rpm until it became completely clear. Biopolymer solutions of fenugreek seed gum and whey protein isolate at different ratios of 1:1, 1:0, and 0:1 were added to the emulsion. The ratio of the biopolymer solution to the emulsion was 70:30. Then, the mixture was homogenized using an ultrathorax homogenizer for 20 min at 18,000 rpm (Delfanian et al., 2018). Encapsulation efficiency, particle size, polydispersity index (PDI), zeta potential, and morphology of nanoemulsions were determined (Esmaeilzadeh Kenari & Razavi, 2022).

#### 2.2.6 Coating of pistachio nuts and storage

Fresh pistachio nuts (without shell and 15% moisture content) were coated by dipping the nuts in the coating solutions for 30 s. Pistachio nuts were air‐dried at room temperature for 1 h and packed in polyethylene bags (75 μm thickness). Packs were kept at 4°C. During an 8‐week period, chemical and microbial tests were performed every 2 weeks. Table 1 shows the code and description of the treatments investigated in this study.

**TABLE 1:** Description of different pistachio samples.

Code of sample	Type of coating	Extract
Control (CONT)	—	—
FSGE	FSG with extract	100 ppm
WPIE	WPI with extract	100 ppm
MIXE	FSG: WPI (with extract)	100 ppm
FSGW	FSG without extract	—
WPIW	WPI without extract	—
MIXW	FSG: WPI (without extract)	—

#### 2.2.7 Pistacho nut tests

The moisture content of pistachio kernels was determined by drying the samples in a vacuum oven (DZF‐6020‐T, Binglin Co., Ltd., Shanghai, China) at 105°C (Tajeddin & Shakerardekani, 2022). The change in color parameters (L*, a*, and b*) of pistachio nuts was measured using the Hunter Lab instrument (LS 5100, Reston, VA) (Gheysarbigi et al., 2020). Peroxide value and thiobarbituric acid value were performed in accordance with the AOCS official method No Cd: 8b‐90 and No Cd: 19‐90, respectively (AOCS, 2017). Fungi development and total mold and yeast count were estimated using the dilution method as described by Razavi et al. (2021). The sensory evaluation was carried out by 10‐trained panelists (7 females/3 males, ages 25–33 years) according to the hedonic method described by Sheikhi et al. (2019). A score of 0–10 was used to evaluate the changes in the sensory properties of pistachio nuts (Sheikhi et al., 2019).

### 2.3 Statistical analysis

The means of the treatments were analyzed according to a completely randomized design. Duncan's multiple range tests at level 0.05% were used to estimate significant differences (*p* < .05). All tests were done in triplicate. SPSS software version 20 was applied to compare the data using analysis of variance (ANOVA).

## 3 RESULTS AND DISCUSION

### 3.1 Bioactive compounds and antioxidant activity of PHE

The total anthocyanin and total phenolic content of the extract were 125.44 ± 2.17 μg cy‐3‐glu/g fw extract and 675.18 ± 3.2 mg GAE/g fw extract, respectively. Rafiee et al. (2017) reported lower phenolic (118.56 μg cy‐3‐glu/g fw extract) and anthocyanin (614.91 mg GAE/g fw extract) content than our results (Rafiee et al., 2017). Phenolic compounds and anthocyanins are common bioactive compounds in pistachio shells. Barreca et al. (2016) reported total phenolic content between 6.74 and 11.7 μM GAE. Also, they reported proanthocyanidins between 0.088 and 0.177 mg CyE/g fw for ethanolic and methanolic extract, respectively (Barreca et al., 2016). The lower total phenolic content reported by Rajaei et al. (2010) was 49.32 mg GAE/g (Rajaei et al., 2010).

**FIGURE 1:**
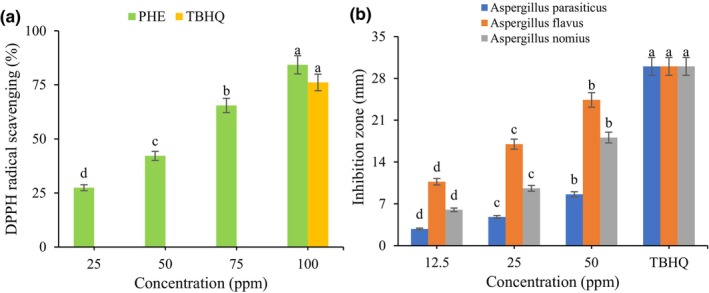
DPPH radical scavenging activity (a) and antifungal activity (b) of PHE.

The results of the DPPH radical scavenging activity of PHE at different concentrations are shown in Figure 1a. As can be seen, the antioxidant activity was increased by an increase in extract concentration, and a statistically significant difference (*p* < .05) was observed. A direct positive correlation emerges between antioxidant activity and the phenolic content of PHE. DPPH and TPC are based on the electron‐donating ability of the antioxidants. Therefore, by increasing the concentration of the PHE, more electron‐donating occurs, and the antioxidant activity increases (Li et al., 2018). The antioxidant activity of PHE has been reported by other researchers (Kazemi et al., 2019; Taghizadeh, Davarynejad, et al., 2018; Taghizadeh, Rezaee, et al., 2018). Barreca et al. (2016) measured the antioxidant activity of PHE by FRAP, DPPH, and ABTS methods. They stated that PHE contains 20 phenolic compounds like gallic acid, hydroxybenzoic acid, protocatechuic acid, and vanillic acid, which exhibit antioxidant activity (Barreca et al., 2016). The antioxidant activity of phenolic compounds is related to the reducing ability of hydroxyl groups linked to phenolic structures and their degree of glycosylation. Seifzadeh et al. (2018) reported IC50 = 4.49 ppm, total phenolic content = 94.79 mg GAE/g DW, and anthocyanin = 0.12 mg/g DW (Seifzadeh et al., 2018). The concentration of 100 ppm of PHE was chosen for encapsulation due to no statistically significant difference (*p* > .05) with the synthetic antioxidant. Different studies have shown a higher or no statistically significant difference between the antioxidant activity of the extract and TBHQ (Basuny et al., 2012; Manzoor et al., 2012; Salami et al., 2020; Taghinia et al., 2019).

### 3.2 Antifungal activity

The results of the inhibition zone of PHE for *Aspergillus flavus*, *Aspergillus parasiticus*, and *Aspergillus nomius* are shown in Figure 1b. All species demonstrated sensitivity to the PHE. An increase in extract concentration caused an increase in the inhibition zone, and a statistically significant difference (*p* < .05) was observed. A reduction in the formation of biofilm revealed a high susceptibility of the toxigenic *Aspergillus* to PHE compounds. Khorasani et al. (2017) measured the antifungal activity of cinnamon, celak, and clove extracts against *Aspergillus flavus*, and their results showed inhibition properties of all extracts (Khorasani et al., 2017). In this study, the sensitivity of *Aspergillus flavus* to the extract was higher than that of *Aspergillus parasiticus* and *Aspergillus nomius*, while Bocate et al. (2019) reported the same inhibition zone for all *Aspergillus* species against silver nanoparticles (Bocate et al., 2019).

### 3.3 Nanoemulsion properties

Nanoemulsions are characterized in terms of particle size, PDI, zeta potential, encapsulation efficiency, and morphology (Carbone et al., 2018). The results of the particle size of different nanoemulsions are illustrated in Table 2. All nanoemulsions have a mean size lower than 265 nm, which demonstrates the proper method of encapsulation. The lower and higher particle sizes were observed in nanoemulsions prepared by WPI and FSG coatings, respectively. This difference is related to the higher emulsifying properties of whey protein isolate, which causes resistance to droplet movements and provides smaller droplets. In previous studies, a particle size lower than 1000 nm was also reported for nanoemulsions of Iranian golpar (Kenari et al., 2020), paper flower (Kenari & Razavi, 2022), and *Fumaria parviflora* (Razavi & Kenari, 2021).

**TABLE 2:** Encapsulation efficiency, particle size, PDI, and zeta potential of different nanoemulsions.

Code of sample	Particle size (nm)	PDI	Zeta potential (mV)	Encapsulation efficiency (EE)
FSGE	264.77 ± 7.6^a^	0.29 ± 0.06^a^	−36.49 ± 1.7^c^	88.12 ± 2.3^a^
WPIE	235.48 ± 6.9^c^	0.15 ± 0.07^c^	−30.13 ± 2.3^a^	67.35 ± 3.1^c^
MIXE	259.03 ± 5.4^b^	0.23 ± 0.05^b^	−44.55 ± 1.2^b^	74.87 ± 4.5^b^

*Note*: Different superscript indicate statistically significant differences at p < 0.05.

Another important parameter that shows the heterogeneity of nanoemulsions is PDI. The PDI of different nanoemulsions ranged from 0.15 to 0.29. PDI below 0.3 is desirable, and in this sense, all samples have optimal PDI. An increase in particle size causes lower PDI and heterogeneity of particles. A similar correlation between PDI and particle size of nanoemulsion was reported by Jafari et al. (2022) for nano and microemulsions of rosemary leaf extract in cress and basil seed gums (Jafari et al., 2022).

Zeta potential is an important factor that provides a description of electrostatic interactions. The zeta potential of nanoemulsions ranged from −30.13 to −44.55 mV (Table 2), which is related to the anionic nature of WPI and FSG. Negative zeta potential of nanoemulsion with native seed gums or whey protein concentration was also reported by other researchers for the extract of paper flower in *Urtica dioica* L. seed gum (Kenari & Razavi, 2022) and garlic in WPI and chitosan (Tavares & Noreña, 2019). Emulsions with a zeta potential between −30 and +30 mV are inherently thermodynamically unstable. Considering that the zeta potential of the nanoemulsions was less than −30 mV, they had good stability.

The encapsulation efficiency of nanoemulsions ranged from 67.35% to 88.12%. The particles with a higher mean size have a higher encapsulation efficiency. Encapsulation efficiency higher than 50% was also reported by other researchers for plant extract encapsulated in different wall materials (Hu et al., 2018; Sarvinehbaghi et al., 2021).

**FIGURE 2:**
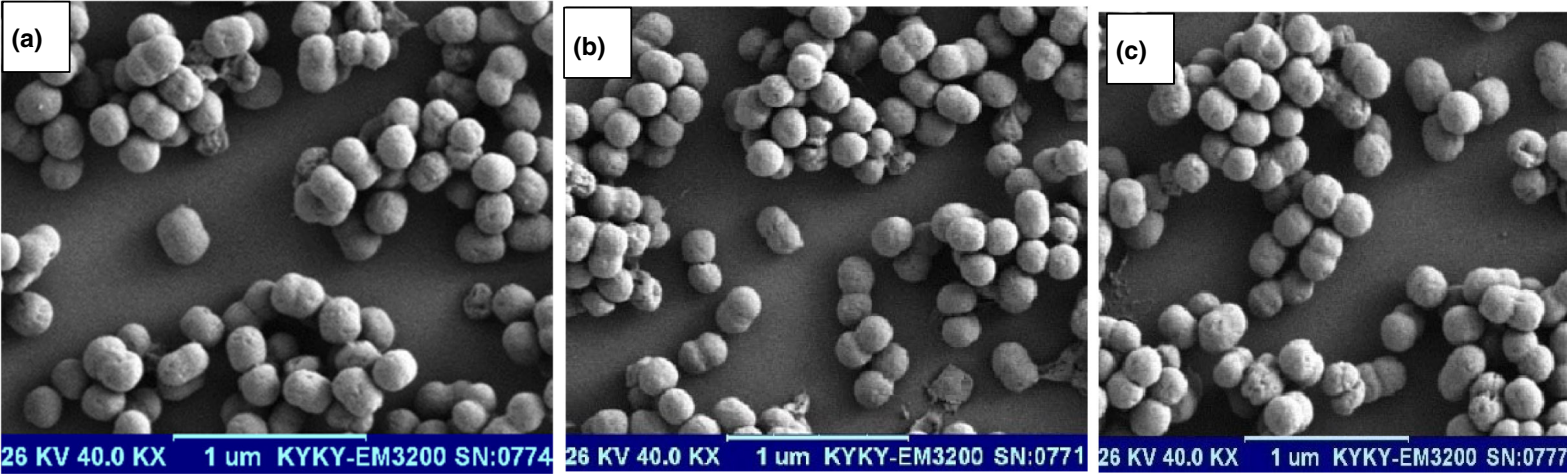
SEM images of different nanoemulsions (a) FSGE, (b) WPIE, and (c) MIXE.

The morphologies of PHE nanocapsules obtained using different wall materials are shown in Figure 2. Generally, all microcapsules had regular spherical shapes, smooth surfaces, and no cracks and dents were observed. These morphological images indicate that the drying stage is done gradually. Ozdemir et al. (2021) evaluated the SEM images of basil essential oil encapsulated in whey protein isolate, maltodextrin, and gum Arabic. They stated no significant difference in the morphologies of microparticles prepared with different coating materials (Ozdemir et al., 2021). Uniformity and minimum agglomeration indicate strong properties of the coating for encapsulating the extract (Kenari & Razavi, 2022). Similar morphologies were observed in the extract encapsulated with protein:carbohydrate coatings (Esmaeilzadeh Kenari & Razavi, 2022; Kenari et al., 2020; Razavi & Kenari, 2021).

### 3.4 Moisture content

Moisture content is one of the important parameters in stored fruits, which is important both economically and qualitatively. The results of the moisture content of different pistachio samples are shown in Figure 2a. The moisture content of pistachio samples decreased significantly (*p* < .05) during storage time. At day 0, no statistically significant difference (*p* > .05) was observed between samples. The control sample had significantly lower moisture content than the coated samples and had a statistically significant difference (*p* < .05) with the other samples. The higher moisture content was observed in the pistachio sample coated with MIX and enriched with PHE. The higher moisture content in coated samples is due to the barrier properties of the coating, which restrict water transfer and inhibit moisture loss. Saeedi et al. (2022) reported an increase in weight loss of pistachio samples stored at 2°C for 45 days due to moisture evaporation (Saeedi et al., 2022). Khoshnoudi‐Nia et al. (2019) reported a higher moisture content in pistachio coated with alginate during storage, which is related to the resistance of gelatin‐coated samples to moisture transmission (Khoshnoudi‐Nia & Sedaghat, 2019). Khajeh‐Ali et al. (2022) evaluated the moisture content of pistachio kernels coated with carboxymethyl cellulose containing Astragalus honey. Their results revealed that coated pistachios had a lower moisture content. The carboxymethyl cellulose coating with and without honey could prevent moisture loss in pistachio samples. The addition of honey increased coating efficiency (Khajeh‐Ali et al., 2022).

### 3.5 Color indexes

**FIGURE 3:**
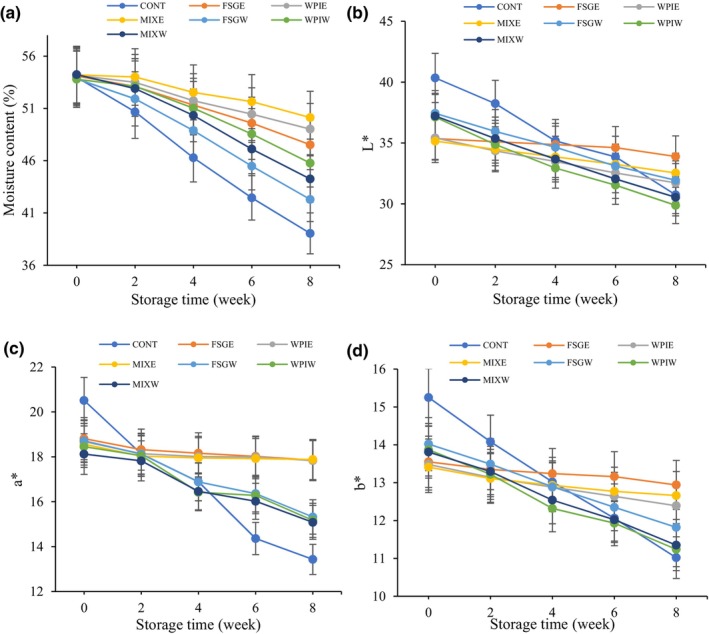
Change in moisture content (a), lightness (b), redness (c), and yellowness (d) of different pistachio samples.

The color change of different pistachio samples stored at 4°C for 8 weeks, as given by lightness (L*), redness (a*), and yellowness (b*), is shown in Figure 3b–d. At day 0, no statistically significant difference (*p* > .05) was observed between the coated samples. The control sample had significantly higher color indexes than the coated samples and had a statistically significant difference (*p* < .05) with other samples. All color indexes were affected by storage time, type of coating, and the use of extract in coatings. Khajeh‐Ali et al. (2022) reported a significant decrease in L* and b* color indexes of pistachio kernels during storage, which is due to chemical and fungi reactions (Khajeh‐Ali et al., 2022). The lower reduction in color parameters was observed in pistachio samples coated with coatings enriched with PHE. It could be attributed to higher membrane integrity, preventing the destruction of pistachio pigments as well as color indexes. These findings were in accordance with those of fresh pistachio kernels. Anthocyanin and chlorophyll are common pigments in pistachio nuts, and they decrease over time (Sheikhi et al., 2019). The presence of PHE in coating materials prevents chlorophyll and anthocyanine decomposition. Inhibition of oxygen contact is also a key factor affecting the stability of color in coated samples during storage (Khoshnoudinia & Sedaghat, 2014).

### 3.6 Oil oxidation

**FIGURE 4:**
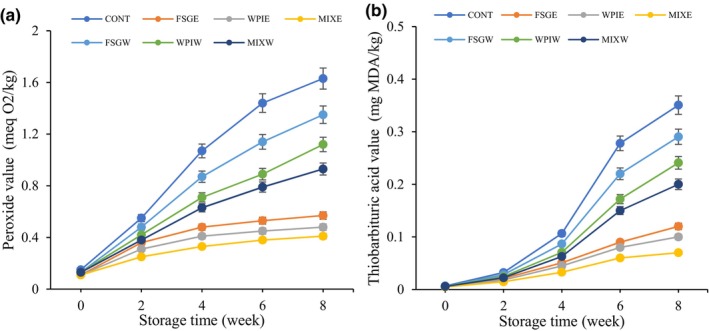
Change in peroxide value (a) and thiobarbituric acid value (b) of different pistachio samples.

PV and TBA are used for the measurement of hydroperoxides and compounds formed during their decomposition. They are the main initial and secondary products of oil oxidation (Kenari & Razavi, 2022). The results of the oil oxidation of different pistachio samples are shown in Figure 4a,b. The rate of oxidation in all samples increased over time, and statistically significant differences (*p* < .05) were observed between different times. Tavakolipour et al. (2020) reported an increasing trend of PV in pistachio powder during storage (Tavakolipour et al., 2020). The control sample showed a higher peroxide value and thiobarbituric acid value than coated samples, and a statistically significant difference (*p* < .05) with other coated samples was observed. Khoshnoudi‐Nia et al. (2019) reported lower PV in roasted pistachio coated with gelatin in comparison to control sample, which is due to the oxygen barrier function of the gelatin coating. Bonilla et al. (2018) reported lower PV in Brazilian and cashew nut coats with gelatin, sodium caseinate, and chitosan than in an uncoated control sample during 120 days of storage at 25°C (Bonilla et al., 2018). A positive correlation has been observed between the antioxidant activity of PHE and the peroxide value, so that the pistachio samples coated with coatings enriched with PHE exhibited lower PV and TBA. At the end of storage time, all samples had a statistically significant difference (*p* < .05). The gradual release of phenolic compounds over a period of time is the main reason for lower oil oxidation in pistachio samples treated with PHE. Roostaee et al. (2017) investigated the antioxidant activity of PHE on the oxidative stability of soybean oil. They stated that the antioxidant activity of PHE is higher than that of BHA and BTH based on peroxide value and thiobarbituric acid value (Roostaee et al., 2017). Sarteshnizi et al. (2021) reported a great efficiency of PHE for controlling lipid oxidation in Sind sardine protein hydrolysates (Sarteshnizi et al., 2021). Strong inhibition of beef patties' lipid oxidation was stated for samples treated with PHE during 8 days of storage (Sadeghinejad et al., 2019). These findings are in accordance with the results of Razavi et al. (2021), who studied the antioxidant capability of thyme extract in a carboxymethyl cellulose coating applied to fresh hazelnuts (Razavi et al., 2021).

### 3.7 Fungi development and total mold and yeast count

**FIGURE 5:**
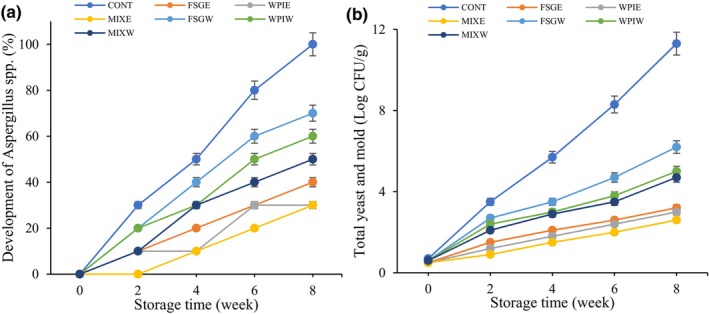
Change in fungi development (a) and total mold and yeast count (b) of different pistachio samples.

The results of fungi development in different samples are shown in Figure 5a. The fungi in all samples developed over time, and statistically significant differences were observed (*p* < .05). The population of yeast and mold significantly (*p* < .05) increased in all samples during storage. The control sample exhibited higher fungi development and total count, and a statistically significant difference (*p* < .05) with other coated samples was observed. The coatings enriched with PHE act as active packaging, which contains antifungal agents, which leads to diminishing fungi growth. Bioactive compounds of PHE interact with microorganism cell walls and change their permeability. Disturbance in the passage of substances led to microorganism death. Moslehi et al. (2021) stated that the content of mold and yeast in pistachio nuts without coating increased during four months of storage, whereas pistachio samples treated with methylcellulose exhibited a lower total count (Moslehi et al., 2021). Other studies also reported that the application of whey protein concentrate with plant extract or carboxymethyl cellulose/gelatin with *Dianthus barbatus* essential oil coatings significantly reduced yeast and mold growth (Mohammadi et al., 2020; Tavakolipour et al., 2011, 2020). Razavi et al. (2021) reported that fungi growth in fresh hazelnut coated with carboxymethyl cellulose and *Thymus vulgaris* extract during 147 days of storage is lower than in untreated samples (Razavi et al., 2021). Similarly, Hashemi et al. (2021) stated that alginate coating enriched with essential oil reduced mold and yeast growth in fresh pistachios (Hashemi et al., 2021).

### 3.8 Sensory properties

The sensory assessments of products have a great influence on consumer acceptance (Hosseini et al., 2019). The results of the sensory attributes of different pistachio samples are illustrated in Table 3. The sensory quality scores for pistachio color, texture, taste, and overall acceptance in all samples decreased during storage. The storage time and PHE in the coating had a significant (*p* < .05) influence on all of the sensory characteristics. No statistically significant difference (*p* < .05) in color was observed between samples in terms of coatings. In the present study, at the end of storage time, pistachio samples with an extract coating exhibited higher sensory scores than other samples. It is because of lower oil oxidation and fungus growth. Hashemi et al. (2021) stated that fresh pistachio coated with gum Arabic and 0.3% *Zataria multiflora Bioss* essential oil showed higher sensory acceptability than the uncoated control sample (Hashemi et al., 2021). As explained about the color indicators, the pigments in pistachio kernels decompose with storage time. The color indexes declined, which has a negative influence on the color sensory score (Saeedi et al., 2022). Khoshnoudi‐Nia et al. (2019) reported a positive correlation between the moisture content and texture of pistachios during storage. According to their research, when the moisture content of the pistachio decreased, the hardness of the texture increased and the texture sensory score was reduced (Khoshnoudi‐Nia & Sedaghat, 2019). A change in the structure of pistachio cell walls occurred as a result of pathogenic activity on the surface of the pistachio, which has a negative effect on the sensory texture score. Other researchers reported that applying a coating with plant essential oil or extract to fresh pistachio and hazelnut leads to higher sensory scores (Boghori et al., 2020; Hashemi et al., 2021; Moslehi et al., 2021).

**TABLE 3:** Sensory scores of fresh pistachio samples during storage.

Sensory properties	Sample	0	2	4	6	8 (week)
Color	CONT	8.8 ± 0.5^Aa^	8.2 ± 0.7^Ba^	7.5 ± 0.9^Cab^	7.0 ± 0.6^Dcd^	6.4 ± 0.9^Ee^
	FSGE	7.8 ± 0.7^Ac^	7.7 ± 0.9^ABb^	7.7 ± 0.4^ABa^	7.6 ± 0.5^Ba^	7.3 ± 0.5^Ca^
	WPIE	7.8 ± 0.8^Ac^	7.6 ± 0.5^ABb^	7.4 ± 0.7^BCbc^	7.3 ± 0.5^Cb^	7.0 ± 0.7^Dbc^
	MIXE	7.8 ± 0.4^Ac^	7.6 ± 0.9^ABb^	7.5 ± 0.5^Bab^	7.4 ± 0.6^Bab^	7.1 ± 0.4^Cab^
	FSGW	8.2 ± 0.5^Ab^	7.8 ± 0.7^Bb^	7.4 ± 0.5^Cbc^	7.2 ± 0.4^Cbc^	6.8 ± 0.8^Dcd^
	WPIW	8.1 ± 0.6^Ab^	7.6 ± 0.6^Bb^	7.2 ± 0.9^Cc^	6.9 ± 0.6^Dd^	6.5 ± 0.6^Ee^
	MIXW	8.0 ± 0.6^Abc^	7.7 ± 0.4^Bb^	7.2 ± 0.5^Cc^	6.9 ± 0.5^Dd^	6.6 ± 0.5^Ede^
Texture	CONT	9.5 ± 0.0^Ab^	7.9 ± 0.5^Bf^	6.7 ± 0.7^Cf^	5.6 ± 0.9^De^	4.2 ± 0.9^Ee^
	FSGE	10.0 ± 0.0^Aa^	8.6 ± 0.6^Bc^	7.9 ± 0.8^Cc^	7.3 ± 0.6^Dbc^	7.0 ± 0.8^Ec^
	WPIE	10.0 ± 0.0^Aa^	9.0 ± 0.0^Bb^	8.3 ± 0.4^Cb^	7.5 ± 0.5^Db^	7.3 ± 0.5^Eb^
	MIXE	10.0 ± 0.0^Aa^	9.3 ± 0.0^Ba^	8.7 ± 0.5^Ca^	8.3 ± 0.8^Da^	7.9 ± 0.4^Ea^
	FSGW	9.5 ± 0.0^Ab^	8.1 ± 0.4^Bef^	7.3 ± 0.7^Ce^	7.1 ± 0.6^Cd^	6.5 ± 0.7^Dd^
	WPIW	9.8 ± 0.0^Ab^	8.2 ± 0.5^Bde^	7.4 ± 0.4^Cde^	7.2 ± 0.4^Ccd^	6.8 ± 0.8^Dc^
	MIXW	10.0 ± 0.0^Aa^	8.4 ± 0.6^Bcd^	7.6 ± 0.9^Cd^	7.3 ± 0.7^Dbc^	6.9 ± 0.7^Ec^
Taste	CONT	10.0 ± 0.0^Aa^	7.9 ± 0.7^Bcd^	6.7 ± 0.5^Ce^	5.6 ± 0.6^De^	4.2 ± 0.9^Ee^
	FSGE	8.4 ± 0.4^Ac^	7.5 ± 0.4^Bf^	7.4 ± 0.8^BCd^	7.2 ± 0.5^CDc^	7.0 ± 0.9^Db^
	WPIE	8.2 ± 0.5^Ac^	7.6 ± 0.8^Bef^	7.5 ± 0.5^BCcd^	7.3 ± 0.4^CDbc^	7.2 ± 0.4^Db^
	MIXE	8.3 ± 0.4^Ac^	7.8 ± 0.5^Bde^	7.7 ± 0.6^BCc^	7.6 ± 0.7^CDa^	7.5 ± 0.6^Da^
	FSGW	9.5 ± 0.0^Ab^	8.1 ± 0.7^Bc^	7.5 ± 0.6^Ccd^	6.5 ± 0.6^Dd^	6.0 ± 0.9^Ed^
	WPIW	9.3 ± 0.4^Ab^	8.6 ± 0.9^Bb^	8.0 ± 0.5^Cb^	7.5 ± 0.8^Dab^	6.5 ± 0.4^Ec^
	MIXW	9.4 ± 0.0^Ab^	9.0 ± 0.0^Ba^	8.5 ± 0.4^Ca^	7.5 ± 0.6^Dab^	6.5 ± 0.5^Ec^
Overall acceptance	CONT	9.5 ± 0.0^Aa^	8.0 ± 0.8^Bb^	6.9 ± 0.5^Cd^	6.0 ± 0.6^Dd^	4.9 ± 0.9^Ed^
	FSGE	8.7 ± 0.4^Ac^	7.9 ± 0.7^Bb^	7.6 ± 0.7^Cbc^	7.3 ± 0.5^Db^	7.1 ± 0.6^Db^
	WPIE	8.6 ± 0.5^Ac^	8.0 ± 0.6^Bb^	7.7 ± 0.8^Cab^	7.3 ± 0.9^Db^	7.2 ± 0.7^Db^
	MIXE	8.7 ± 0.9^Ac^	8.2 ± 0.7^Ba^	7.9 ± 0.6^Ca^	7.7 ± 0.6^CDa^	7.5 ± 0.7^Da^
	FSGW	9.0 ± 0.0^Ab^	8.0 ± 0.7^Bb^	7.4 ± 0.5^Cc^	6.9 ± 0.8^Dc^	6.4 ± 0.7^Ec^
	WPIW	9.0 ± 0.0^Ab^	8.1 ± 0.8^Bab^	7.5 ± 0.6^Cbc^	7.2 ± 0.7^Db^	6.6 ± 0.9^Ec^
	MIXW	9.1 ± 0.0^Ab^	8.3 ± 0.5^Ba^	7.7 ± 0.6^Cab^	7.2 ± 0.6^Db^	6.6 ± 0.5^Ec^

*Note*: Upper case letters in each raw indicate the statistically significant difference between days. Lower case letters in each raw indicate the statistically significant difference between samples.

## 4 CONCLUSION

In this study, we carried out an antioxidant and antifungal activity of pistachio hull extract (PHE) on the shelf life extension of fresh pistachio nuts. The results revealed that PHE is a rich source of phenolic compounds (total anthocyanin and total phenolic content of the extract were 125.44 ± 2.17 μg cy‐3‐glu/g fw extract and 675.18 ± 3.2 mg GAE/g fw extract, respectively) with antioxidant and antifungal activities. The use of WPI and FSG composite coatings for nanoencapsulation of PHE is an effective way to increase the performance of PHE as a preservative. The gradual release of phenolic compounds from the coating and the reduction in the penetration of oxygen and moisture into the pistachios during storage are the main reasons for the increased shelf life of encapsulated samples compared to the control. A higher sensory score was observed in samples coated with composite coatings containing PHE than in other samples. At the end of storage time, the overall acceptance of coated samples was higher than 6.4, indicating the efficiency of coating on the sensorial properties of fresh pistachio. The study's results suggest using MIXE as a natural and functional coating on fresh pistachio nuts. Future studies can be focused on the efficiency of complex coatings of other proteins and native seed gums to encapsulate PHE and its application to other fresh nuts.

## AUTHOR CONTRIBUTIONS

**Ali Fatemi:** Formal analysis (equal); methodology (equal); writing – original draft (equal). **Ali Najafi:** Conceptualization (equal); formal analysis (equal); investigation (equal); methodology (equal); project administration (lead); resources (equal); supervision (lead); writing – original draft (equal); writing – review and editing (equal). **Razie Razavi:** Conceptualization (equal); formal analysis (equal); investigation (equal); methodology (equal); software (equal); visualization (equal); writing – original draft (equal); writing – review and editing (equal). **Shima Jafarzadeh:** Investigation (equal); validation (equal); visualization (equal); writing – review and editing (equal).

## FUNDING INFORMATION

None.

## CONFLICT OF INTEREST STATEMENT

The authors have declared no conflict of interest.

## ETHICS STATEMENT

This article does not contain any studies with human or animal subjects.

## Data Availability

Data will be made available on request.
